# Measuring the robustness of link prediction algorithms under noisy environment

**DOI:** 10.1038/srep18881

**Published:** 2016-01-06

**Authors:** Peng Zhang, Xiang Wang, Futian Wang, An Zeng, Jinghua Xiao

**Affiliations:** 1 School of Science, Beijing University of Posts and Telecommunications, Beijing 100876, P.R. China; 2 School of Systems Science, Beijing Normal University, Beijing 100875, P.R. China

## Abstract

Link prediction in complex networks is to estimate the likelihood of two nodes to interact with each other in the future. As this problem has applications in a large number of real systems, many link prediction methods have been proposed. However, the validation of these methods is so far mainly conducted in the assumed noise-free networks. Therefore, we still miss a clear understanding of how the prediction results would be affected if the observed network data is no longer accurate. In this paper, we comprehensively study the robustness of the existing link prediction algorithms in the real networks where some links are missing, fake or swapped with other links. We find that missing links are more destructive than fake and swapped links for prediction accuracy. An index is proposed to quantify the robustness of the link prediction methods. Among the twenty-two studied link prediction methods, we find that though some methods have low prediction accuracy, they tend to perform reliably in the “*noisy*” environment.

The increasing availability of data has helped us largely deepen our understanding of many real systems^1^,^2^,^3^,^4^,^5^,^6^, as well as make predictions^4^. For example, after the *American Physical Society* (APS) citation data was open to public for free download, many interesting phenomena such as the decay of fitness^7^, the inheritance of scientific memes^8^ have been discovered. In addition, the prediction of individual paper’s citation number is also done with the *z*-score based method^9^ and a mechanistic model^10^. Examples can also be found in many other fields, ranging from biological to social systems^11^. The publication of the online user rating data by *Grouplens*^12^ and *Netflix*^13^ has led to hundreds of new recommendation algorithms^14^. However, as the data size is getting bigger and bigger, the quality of the gathered data becomes a serious concern for many researchers. Due to the mistakes in real data, many empirically observed phenomena might turn out to be fake^15^.

The rich real data evoke a very active research field called “complex networks”^16^. Link prediction is a microscopic prediction in complex network^11^. Instead of predicting the collective properties such as degree, clustering coefficient or mean shortest path length, it aims to estimate the likelihood of two nodes to interact with each other in the future, based on the observed network structure^17^. Such research topic is strongly connected to many other fields such as online product recommendation^18^, biological network reconstruction^19^ and community detection^20^. Due to the wide applications, many link prediction methods have been proposed recently. The existing methods can be divided into three categories: node-based similarity algorithm^11^, path-based similarity algorithm^21^ and Bayesian estimation algorithm^22^. Some of these methods have been applied to identify both missing and spurious interactions in networks^23^,^24^.

So far, the validation of these link prediction algorithms are usually done within the framework of training set (observed network) and testing set (future network) data division^25^. The algorithms are run on the training set while the testing set is used to measure the accuracy of the prediction. In most link prediction studies, the training set is assumed to be entirely clean^11^. However, in real cases the reliability of observed network data is not always guaranteed. For instance, biological networks that are inferred from experiments or social networks that result from spontaneous human activity may contain inaccurate and misleading information, resulting in missing and spurious links^26^. Therefore, when applied to solve real problems, the link prediction methods will most likely work under a noisy environment. Even though many tests and comparisons with random and null models are executed in the literature for avoiding common errors^27^,^28^,^29^, how the prediction results would be affected if the observed network data is unclean has not yet been fully understood^11^.

In this paper, we investigate the robustness of the existing link prediction algorithms in the real networks where some links are missing, fake or swapped with other links. Both random noise and biased noise in the observed link data are considered. In order to quantify and compare the robustness of different link prediction algorithms, an index is proposed in this paper. It computes the area under the prediction accuracy curve with different fraction of noisy data. By using this robustness index, we find that though some methods have low prediction accuracy, they tend to perform reliably in the “noisy” environment. Our results highlight that the performance of a link prediction algorithm should not only be judged by its accuracy but also by its reliability. This new idea may inspire the design of some new link prediction methods with high performance in both aspects.

## Results

Let us consider an undirected network *G*(*V*, *E*) where *V* is the node set and *E* is the link set. In link prediction problem, *E* is divided into a training set *E*^*T*^ and a probe set *E*^*P*^. Usually, 90% of the links in real networks are put in *E*^*T*^ and the remaining 10% forms *E*^*P*^. The training set is treated as the observed network and the link prediction algorithms will run on it. The links in the testing set are considered as the future or missing links. They will be used to examine the accuracy of the link prediction algorithms. The prediction accuracy is measured by the standard metric of the area under the receiver operating characteristic curve (AUC), see definition in the Method section. There are many existing link prediction algorithms in the literature. In this paper, we consider 22 link prediction methods. The results in the main paper will be based on three representative ones: Common Neighbors (CN)^30^, Jaccard^30^ and Resource Allocation (RA)^31^ (see the Method section for the detail of these methods). CN is the most straightforward algorithm for link prediction. It simply calculates the similarity between nodes by counting the number of common neighboring nodes. Jaccard can reduce the bias of CN to large degree nodes, and RA is one of the best performing link prediction algorithms in accuracy. Due to the page limit, the results of other 19 methods are reported in the Supplementary Material (SI).

The real networks we considered in this paper are: USAir (Airline network)^11^, Political blogs (hyper links between blogs)^32^, Jazz (friendship network)^33^, C.elegans (neural network)^34^, E.coli (metabolic network)^35^, Netsci (scientific collaboration network)^30^, Email (email contact network)^36^, TAP (protein-protein interaction network)^37^, Word (noun-adjectives adjacency network)^30^, Dolphins (friendship network)^38^. The basic properties of these networks are shown in Table 1. Throughout the paper, we mainly show the results of C.elegans, Jazz, USAir and PB by figures as examples. The results of the rest networks are presented in Fig. 3 (the exact numbers of the results in Fig. 3 are summarized in a table and reported in SI).

We now describe the model we proposed to study the performance of the link prediction algorithms under noisy environment. First, we consider the cases where some random noise exists in the observed networks. In particular, after the real network is divided into the training set *E*^*T*^ and probe set *E*^*P*^, some links are randomly added to or deleted from *E*^*T*^. We define a quantity *ratio* to measure the fraction of randomly added or deleted links. When *ratio* is positive, |*ratio*|*|*E*^*T*^| links are randomly added to the training set. When *ratio* is negative, |*ratio*|*|*E*^*T*^| links are randomly deleted from the training set. In order to keep the network connected, we cannot remove too many links from *E*^*T*^. Therefore, we keep −40% ≤ *ratio* ≤ 100%. We have to emphasize that the link deletion process considered in our paper is not equivalent to simply reducing the training set. In link prediction problem, the whole data set is divided into two parts, the training set and the probe set (e.g. 90% vs 10%). In the case of link deletion, the probe set is fixed but some links in the training set are randomly removed. In the case of reducing the training set, the links reduced in the training set are moved to the probe set (e.g. 80% vs 20% data division). In this case, the probe set is larger, so that it is easier to predict the missing links.

**Figure 1:**
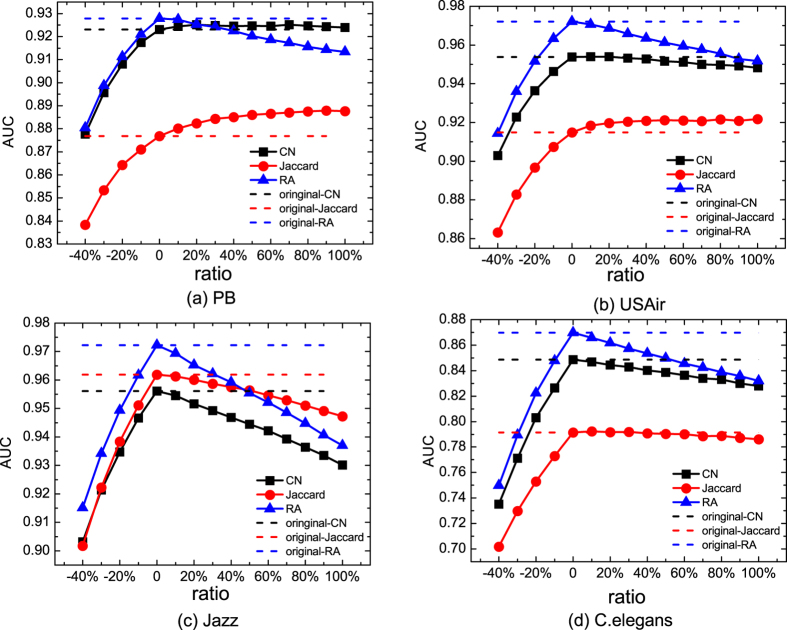
The dependence of link prediction algorithms’ accuracy (AUC) on the fraction of missing and noisy links in four real-world networks. The four networks are PB, USAir, Jazz and C.elegans. The link prediction algorithms are *CN*, *Jaccard* and *RA*. Dashed lines represent the AUC of these prediction algorithms in the clean network (i.e. link prediction AUC based on the network without any noisy or missing links). *ratio* < 0 represents the missing link case and *ratio* > 0 stands for the noisy link case.

In Fig. 1, we show the effect of random noise on the link prediction results. More precisely, we investigate the dependence of *AUC* on |*ratio*|. One immediate observation is that *AUC* in general decreases with |*ratio*|. However, in some networks like PB and USAir, adding some noisy links can improve the accuracy of the Jaccard method. This phenomenon is similar to the results found in ref. 39 where the recommendation accuracy can be improved by adding some virtual links. The underlying reason for this is that the random links improve the connectivity of the network, making the similarity matrix denser. Therefore, many missing links that were impossible to predict (due to the low connectivity) becomes predictable with these randomly added links. Another observation in this figure is that given one link prediction method, randomly removing links are more destructive than adding random links given the same |*ratio*| value. Taking the CN method in PB network as an example, removing 40% links will decrease *AUC* from 0.92 to 0.88. However, adding the same amount of random links will make *AUC* stay around 0.92.

Another feature shown in Fig. 1 is that *AUC* of these link prediction algorithms decays with |*ratio*| with different speed. Taking the C.elegans network as an example, *AUC* of all the link prediction methods decreases with |*ratio*|. Even though RA has the highest *AUC* when *ratio* = 0, the *AUC* of RA decreases fastest. When *ratio* = 100%, the *AUC* of RA is almost the same as that of CN. In the Jazz network, the *AUC* of RA decays so fast that it is even lower than the *AUC* of the Jaccard method when *ratio* = 100%. This indicates that when noise exists in the network, the performance of different link prediction methods may change dramatically. However, such effect is not prominent in all networks. For instance, the crossover point between Jaccard and CN does not exist in PB, C.elegans and USAir networks. Therefore, we need to develop a way to quantify the different decay speed of *AUC*.

We propose a metric called “algorithm robustness” to quantify to what degree the link prediction algorithms can resist the noise in the observed network. Mathematically, it reads


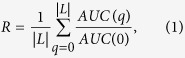


where *L* = ratio*|*E*^*T*^|, and *AUC*(*q*) is the *AUC* of a link prediction method when *q* links are added to the observed network. Note that we define *R* = 1 when *ratio* = 0. *R* can also be used to measure the algorithm robustness when links are removed from the training set. Apparently, *R* depends on *ratio*. We first fix *ratio* = −40% and 40%, and compare the robustness of different algorithms in real networks in Fig. 3. In this figure, *R*^+^ is the *R* value when 40% random links are added to the training set. Similarly, *R*^−^ is the *R* value when 40% links are randomly deleted from the training set. From Fig. 3, one can see that RA has the highest *AUC* value, indicating that this method is the best performing method by traditional definition of the link prediction problem. However, when noise exists, the *R* value of RA becomes lowest among these three methods. This is because RA is very sensitive to noise, so its *AUC* decays very fast with *ratio*. The Jaccard method has a relatively lower *AUC* than RA, but its *R* is much higher than RA. These results indicate that Jaccard is a more reliable link prediction algorithm than RA.

**Figure 2:**
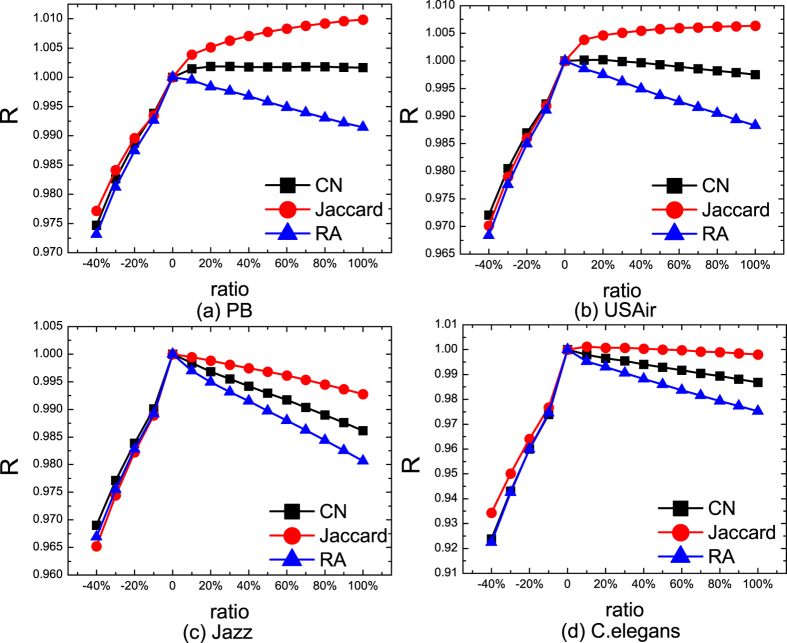
The dependence of the robustness of the algorithms (R) on the fraction of missing and noisy links in four real-world networks.

We then move to investigate the effect of *ratio* on *R*. The results are shown in Fig. 2. We can see that when *ratio* is positive, the difference between algorithms’ *R* increases with *ratio*. The order of *R* of these method does not change with *ratio*. This indicates that using a particular *ratio* to calculate *R* is sufficient to compare the robustness of algorithms in different *ratio*.

**Figure 3:**
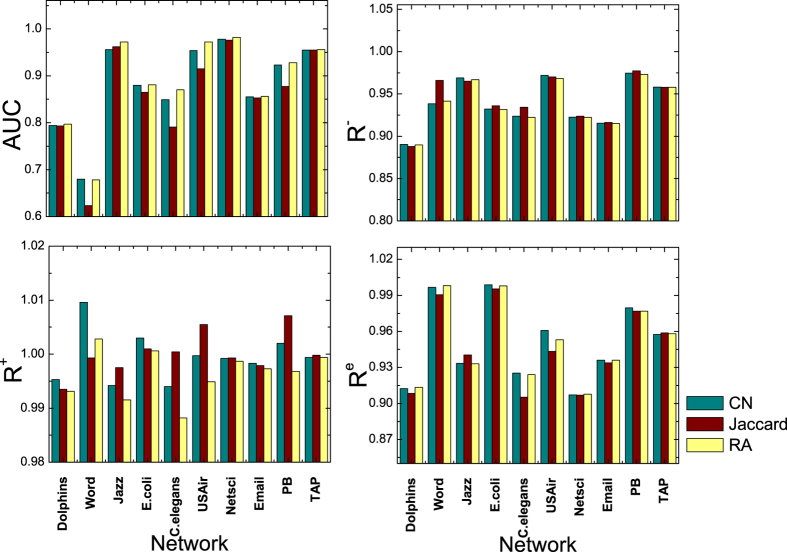
The *AUC* and robustness of link prediction algorithms in ten real networks. *R*^−^, *R*^+^ and *R*^*e*^ are respectively the robustness of the algorithms with missing links, noisy links and swapped links. The fraction of changed links here is 40%.

Besides randomly adding and deleting links, we also consider the link swapping procedure to simulate the noise in real networks. In each step, we randomly pick up two links, *a*-*b* and *c*-*d*. Then we swap the link as *a*-*d* and *c*-*b*. In this way, we can preserve the node degree but randomize the network. This procedure models the noise that does not influence the node degree but alter the detailed connections in the network. To be consistent with the noise model above, we also use *ratio* = 40% here to compute *R*, denoted as *R*^*e*^. The difference is that here we carry out the swapping for ratio*|*E*^*T*^| steps. The results of *R*^*e*^ is reported in Fig. 3. One can see that *R*^*e*^ of RA is no longer significantly lower than the other two, indicating that the drawback of RA is its sensitivity to the change of degree distribution of the network.

To support our findings above, we carry out some additional tests. Firstly, there is another way of link prediction evaluation which measures the area under the Precision-Recall curve (AUPR). With AUPR, we can also define the “algorithm robustness” as 
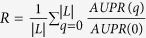
. The results of the algorithm robustness based on *AUPR* are shown in Fig. S1 and S2. One can see that the results with *AUPR* are qualitatively consistent with the results with *AUC* (i.e. randomly removing links are more destructive than adding random links). Meanwhile, one can also notice that the advantage of Jaccard algorithm seems to be more prominent with *AUPR*. Secondly, we tested the link prediction methods under 80% and 50% training set. The results are respectively shown in Fig. S3 and S4. One can see that Jaccard still has higher robustness than RA and CN when training set is 80%. However, it cannot maintain higher robustness when the training set is sparse (i.e. 50%). This is reasonable because when the links in the training set are few, the information for predicting missing links is very limited and most of the link prediction methods will perform equally bad. Thirdly, we perform the *n*-fold cross validation of our experiments. Here, we set *n* = 10. That is, we divide the data into 10 subsets with equal size. Each single subset is retained as the validation data for testing the method, and the remaining 9 subsets are used as training data. The results obtained from the 10-fold cross validation are shown in Fig. S5

To further understand the effect of the change of the degree distribution on link prediction, we study the biased disturbance on four real networks. We introduce a parameter *θ* to control the preference of the noisy links and missing links connecting to nodes with different degrees. More specifically, the links are no longer added or deleted randomly, but biased to node degrees. When a link is about to add to the observed network, the first node will be randomly picked from the network. We then compute the probability that a node will be selected as the second node to receive this link. The probability is


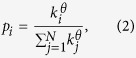


where *θ* is a tunable parameter. When *θ* = 0, the noise is unbiased. When *θ* > 0, the nodes with larger degree tend to receive/cut more links, and vice versa.

**Figure 4:**
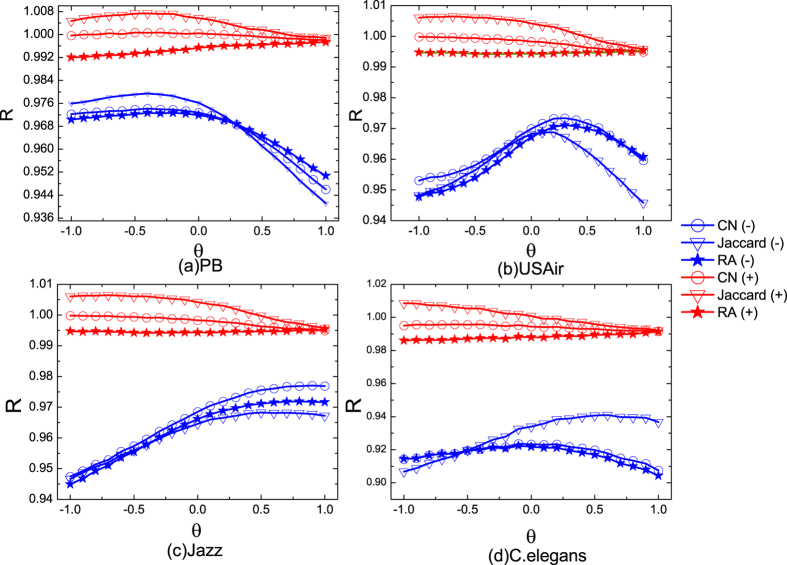
The effect of different biased disturbance on four real-world networks. The tunable parameter *θ* is used to control the preference of the noisy links and missing links connecting to nodes with different degree. The red curves are the results of *R*^+^ while the blue curves are the results of *R*^−^.

The results of the biased noise are shown in Fig. 4. We first discuss the case where links are biased added. In general, *R* of Jaccard is larger than that of CN and RA. The advantage of it becomes bigger when *θ* < 0. This is in fact very important, as in real systems (especially in online systems and citation systems) many malicious attacks tend to add links to the small degree nodes in order to push up their popularity. The Jaccard method can outperform the other two in resisting these malicious attacks. When link removal is biased, *θ* < 0 is generally more destructive. This is because many links of small degree nodes will be further removed, making the missing links of these nodes more difficult to be predicted. Interestingly, we also find that when *θ* = 1, the *R* of all three methods becomes low as well in PB and USAir. This is because removing the links of the large degree nodes will strongly reduce their similarity to other nodes. Even though these large degree nodes will have many links in the future, the link prediction algorithms will fail to predict them as the similarity of the large degree nodes and other nodes becomes too small. Taken together, the bias of noisy links and missing links may further decrease the accuracy of the link prediction algorithms, making it more necessary to consider the robustness of the algorithms in the networks where the source network data is not clean.

With the algorithm robustness index, we re-examine most of the link prediction algorithms. Besides the CN, Jaccard and RA methods, we consider 19 more methods. The results are shown in the SI. In general, the algorithms with high accuracy tend to have low robustness in *R*^+^ and *R*^*e*^ (with some small difference from one method to another). However, the *R*^−^ of the high-accuracy link prediction algorithms can vary significantly. The best selection of the link prediction methods in different cases is discussed in the SI.

## Discussion

Predicting the missing or future links is a very important research topic itself and has applications in many different domains. Although many link prediction methods have been proposed in the literature, the validation of these methods is so far mainly conducted in the assumed noise-free networks and we still miss a clear understanding of how the results would be affected if the observed network data is no longer accurate. In this paper, we study the robustness of the existing link prediction algorithms in the real networks where some links are missing, fake or swapped with other links. We propose an index to quantify the robustness of the link prediction methods. The results show that though some methods have low prediction accuracy, they tend to perform reliably in the “noisy” environment. In addition to accuracy, our work opens up another dimension for studying the link prediction problem.

Our paper raises up some questions for future research. The robustness of the link prediction algorithms in the real networks would be an important evaluation standard as the accuracy. An interesting question would be finding a new method with both high accuracy and strong robustness. In addition, one can use the link prediction to “clean” the noisy environment (i.e. to remove the unreliable links), then finally improve the prediction accuracy for the future links. This is actually also an interesting direction which asks for future study.

## Methods

The link prediction algorithms we used in this paper: common neighbors (CN), Jaccard and resource allocation (RA). We denote the set of neighbors of node *x* by Γ(*x*). For node pair (*x*, *y*), the set of their common neighbors is denoted as 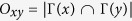. *k*_*z*_ is the degree of node *z*. (1) Common neighbors (CN). In common sense, two nodes are more likely to form a link if they have more common neighbors. The simplest measure of the neighborhood overlap is the direct count,


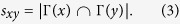


(2) Jaccard. This index was proposed by Jaccard over a hundred years ago, it has a similar definition, related to the probability of triangles in all connected links of any two nodes, which is defined as


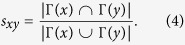


(3) Resource allocation (RA). In this algorithm, the weight of the neighboring node is negatively proportional to its degree. The score is thus denoted as


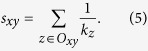


The *AUC* (Area Under the ROC Curve) is a way to quantify the accuracy of prediction algorithms^40^. We denoted the final prediction score sequence of all non-existing links in the training set as *S*. Then, at each time we randomly select a true non-existing link in original network and a link in the probe set to compare their position in the sequence of *S*. Assuming that there are *n*′ times the score of the probe link is higher and *n*″ times two links are with the same score *S*, then the *AUC* value is computed as


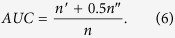


## Additional Information

**How to cite this article**: Zhang, P. *et al*. Measuring the robustness of link prediction algorithms under noisy environment. *Sci. Rep*. **6**, 18881; doi: 10.1038/srep18881 (2016).

## Supplementary Material

Supplementary Information

## Figures and Tables

**Table 1:** The structure properties of ten real networks.

Properties	Dolphins	Word	Jazz	E.coli	C.elegans	USAir	Netsci	Email	PB	TAP
*N*	62	112	198	230	290	332	379	1133	1222	1373
*E*	159	425	2742	695	2148	2126	914	5451	16714	6833
*H*	1.33	1.82	1.39	2.37	1.80	3.46	1.66	1.94	2.97	1.64

Structure properties include network size (*N*), edge number (*E*), degree heterogeneity (*H*). The degree heterogeneity is defined as *H* = 〈*k*^2^〉/〈*k*〉^2^ where *k* is the degree sequence of nodes in the network.

## References

[b1] Dorogovtsev S. N. & Mendes J. F. Evolution of networks. Adv. Phys. 51, 1079 (2002).

[b2] Boccaletti S., Latora V., Moreno Y., Chavez M. & Hwang D. U. Complex networks: structure and dynamics. Phys. Rep. 424, 175–308 (2006).

[b3] Costa L. D. F., Rodrigues F. A., Travieso G. & Villas Boas P. R. Characterization of complex networks: a survey of measurements. Adv. Phys. 56, 167–242 (2007).

[b4] Getoor L. & Diehl C. P. Link Mining: A Survey. ACM SIGKDD Explorations Newsletter 7, 3–12 (2005).

[b5] Barabási A. L. The network takeover. Nat. Phys. 8, 14 (2011).

[b6] Kitsak M. . Identification of influential spreaders in complex networks. Nat. Phys. 6, 888–893 (2010).

[b7] Medo M., Cimini G. & Gualdi S. Temporal Effects in the Growth of Networks. Phys. Rev. Lett. 107, 238701 (2011). 22182132 10.1103/PhysRevLett.107.238701

[b8] Kuhn T., Perc M. & Helbing D. Inheritance patterns in citation networks reveal scientific memes. Phys. Rev. X 4, 041036 (2014).

[b9] Gualdi S., Medo M. & Zhang Y. C. Influence, originality and similarity in directed acyclic graphs. Europhys. Lett. 96, 18004 (2011).

[b10] Thomas S. M. & Beierkuhnlein C. Predicting ectotherm disease vector spreadBenefits from multidisciplinary approaches and directions forward. Naturwissenschaften 100, 395–405 (2013). 23532546 10.1007/s00114-013-1039-0

[b11] Lü L. & Zhou T. Link prediction in complex networks: A survey. Physica A 390, 1150–1170 (2011).

[b12] Konstan J. A. . GroupLens: applying collaborative filtering to Usenet news. Communications of the ACM 40, 77–87 (1997).

[b13] Bell R. M. & Koren Y. Lessons from the Netflix prize challenge. ACM SIGKDD Explorations Newsletter 9, 75–79 (2007).

[b14] Sun D. . Information filtering based on transferring similarity. Phys. Rev. E 80, 017101 (2009). 10.1103/PhysRevE.80.017101 19658838

[b15] Butts C. T. Network inference, error, and informant (in) accuracy: a Bayesian approach. Soc. Networks 25, 103–140 (2003).

[b16] Albert R. & Barabási A. L. Statistics mechanics of complex networks. Rev. Mod. Phys 74, 47 (2002).

[b17] Lin D. An information-theoretic definition of similarity. in *Proceedings of the 15th International Conference on Machine Learning*, 296–304 (Madison, Wisconsin, USA, 1998).

[b18] Linden G., Smith B. & York J. Amazon. com recommendations: Item-to-item collaborative filtering. IEEE, Internet Comput. 7, 76–80 (2003).

[b19] Herrgrd M. J. . A consensus yeast metabolic network reconstruction obtained from a community approach to systems biology. Nat. Biotechnol. 26, 1155–1160 (2008). 18846089 10.1038/nbt1492 PMC4018421

[b20] Radicchi F. . Defining and identifying communities in networks. P. Natl. Acad. Sci. USA 101, 2658–2663 (2004). 10.1073/pnas.0400054101 PMC365677 14981240

[b21] Lü L., Jin C. H. & Zhou T. Similarity index based on local paths for link prediction of complex networks. Phys. Rev. E 80, 046122 (2009). 10.1103/PhysRevE.80.046122 19905405

[b22] Liu Z., Zhang Q. M., L. L. & Zhou T. Link prediction in complex networks: A local naive Bayes model. Europhys. Lett. 96, 48007 (2011).

[b23] Zhang P., Zeng A. & Fan Y. Identifying missing and spurious connections via the bi-directional diffusion on bipartite networks. Phys. Lett. A 378, 2350–2354 (2014).

[b24] Guimerà R. & Sales-Pardo M. Missing and spurious interactions and the reconstruction of complex networks. P. Natl. Acad. Sci. USA 106, 22073 (2009). 10.1073/pnas.0908366106 PMC2799723 20018705

[b25] Zhu Y. X., Lü L., Zhang Q. M. & Zhou T. Uncovering missing links with cold ends. Physica A 391, 5769–5778 (2012).

[b26] Costa L. D. F. . Analyzing and modeling real-world phenomena with complex networks: a survey of applications. Adv. Phys. 60, 329–412 (2011).

[b27] Libe-Nowell D. & Kleinberg J. The link prediction problem for social networks. *In Proceedings of the twelfth international conference on Information and knowledge management* (CIKM ‘03). ACM, New York, NY, USA, 556–559 (2003).

[b28] Aiello L. M. . Friendship prediction and homophily in social media. ACM Trans. Web 6, 373–382 (2012).

[b29] Aiello L. M., Barrat A., Cattuto C., Schifanella R. & Ruffo G. Link creation and information spreading over social and communication ties in an interest-based online social network. EPJ Data Sci. 1, 1–31 (2012).

[b30] Newman M. E. Finding community structure in networks using the eigenvectors of matrices. Phys. Rev. E 74, 036104 (2006). 10.1103/PhysRevE.74.036104 17025705

[b31] Zhou T., Lü L. & Zhang Y. C. Predicting missing links via local information. Eur. Phys. J. B. 71, 623 (2009).

[b32] Adamic L. A. & Glance N. The Political Blogosphere and the 2004 U.S. Election: Divided They Blog. in *Proceedings of the 3rd international workshop on Link discovery* 36–43 (New York, NY, USA, 2005).

[b33] Gleiser P. M. & Danon L. Community structure in jazz. Adv. complex syst. 6, 565–573 (2003).

[b34] Watts D. J. & Strogatz S. H. Collective dynamics of ‘small-word’ networks. Nature 393, 440–442 (1998). 9623998 10.1038/30918

[b35] Jeong H., Tombor B., Albert R. . The large-scale organization of metabolic networks. Nature 407, 651–654 (2000). 11034217 10.1038/35036627

[b36] Guimera R., Danon L., Diaz-Guilera A., Giralt F. & Arenas A. Self-similar community structure in a network of human interactions. Phys. Rev. E 68, 065103 (2003). 10.1103/PhysRevE.68.065103 14754250

[b37] Gavin A. C., Bösche M., Krause R. . Functional organization of the yeast proteome by systematic analysis of protein complexes. Nature 415, 141–147 (2002). 11805826 10.1038/415141a

[b38] Newman M. E. & Girvan M. Finding and evaluating community structure in networks. Phys. Rev. E 69, 026113 (2004). 10.1103/PhysRevE.69.026113 14995526

[b39] Zhang F. & Zeng A. Improving information filtering via network manipulation. Europhys. Lett. 100, 58005 (2012).

[b40] Fawcett T. An introduction to ROC analysis. Pattern. Recogn. Lett. 27, 861 (2006).

