# Supplementary material for: Measuring the robustness of link prediction algorithms under noisy environment
Source: Sci Rep. 2016 Jan 6;6:18881. doi: 10.1038/srep18881 (PMC4702065; doi:10.1038/srep18881)
Supplement: Supplementary Information [file srep18881-s1.pdf]

# Supplementary Information of "Measuring the robustness of link prediction algorithms under noisy environment"

Peng Zhang, Xiang Wang, Futian Wang, An Zeng and Jinghua Xiao

## I. METHOD DESCRIPTION

In this paper, we consider 22 link prediction methods. The Common Neighbor (CN), Jaccard and Resource Allocation (RA) methods are described in the paper. Here we describe the rest of methods. They are LocalPath (LP) [1], Katz [2],Salton [3], Sorenson [4],Hub Depressed Index(HDI) [5], Hub Promoted Index (HPI)[6], Leicht-Holme-Newman similarity (LHN)[7], Adamic-Adar(AA)[8], Preferential Attachment(PA)[9], Local Naive Bayes form of CN(LNBCN)[10], Local Naive Bayes form of RA(LNBRA)[10], Leicht-Holme-Newman Index(LHNII)[7], Average Commute Time(ACT) [11],Cosine based on $L^{+}$(CosPlus)[12], Random Walk with Restart(RWR)[13], Local Random Walk(LRW)[14], Superposed Random Walk(SRW)[14], Matrix Forest Index(MFI)[15], CN based on transferring similarity (TSCN)[16].

(1)Local Path Index (LP). To provide a good tradeoff of accuracy and computational complexity, we here introduce an index that takes consideration of local paths, with wider horizon than CN. It is defined as

$$S^{LP} = A^2 + \epsilon A^3, \tag{1}$$

where $\epsilon$ is a free parameter.

(2)Katz Index. This index is based on the ensemble of all paths, which directly sums over the collection of paths and is exponentially damped by length to give the shorter paths more weights. The mathematical expression reads as

$$S_{xy}^{Katz} = \sum_{l=1}^{\infty} \beta^l \cdot |paths_{xy}^{<l>}|, \tag{2}$$

where $paths_{xy}^{<l>}|$ is the set of all paths with length l connecting x and y, and $\beta$ is a free parameter (i.e., the damping factor) controlling the path weights.

(3)Salton Index. It is defined as

$$S_{xy}^{Salton} = \frac{|\Gamma(x) \cap \Gamma(y)|}{\sqrt{k_x \times k_y}}, \tag{3}$$

where $k_x$ is the degree of node x. The Salton index is also called the cosine similarity in the literature.

(4)Søensen Index. This index is used mainly for ecological community data, and is defined as

$$S_{xy}^{S\o renson} = \frac{2|\mathit{\Gamma}(x) \cap \mathit{\Gamma}(y)|}{k_x \times k_y}, \tag{4}$$

(5)Hub Depressed Index (HDI). Analogously to the above index, we also consider a measurement with the opposite effect on hubs, defined as

$$S_{xy}^{HDI} = \frac{|\mathit{\Gamma}(x) \cap \mathit{\Gamma}(y)|}{max\{k_x, k_y\}}, \tag{5}$$

(6)Hub Promoted Index (HPI). This index is proposed for quantifying the topological overlap of pairs of substrates in metabolic networks, and is defined as

$$S_{xy}^{HPI} = \frac{|\mathit{\Gamma}(x) \cap \mathit{\Gamma}(y)|}{min\{k_x, k_y\}}, \tag{6}$$

Under this measurement, the links adjacent to hubs are likely to be assigned high scores since the denominator is determined by the lower degree only. (7)Leicht-Holme-Newman Index (LHN1). This index assigns high similarity to node pairs that have many common neighbors compared not to the possible maximum, but to the expected number of such neighbors. It is defined as

$$S_{xy}^{LHN1} = \frac{|\mathit{\Gamma}(x) \cap \mathit{\Gamma}(y)|}{k_x \times k_y}, \tag{7}$$

(8)Adamic-Adar Index (AA). This index refines the simple counting of common neighbors by assigning the less-connected neighbors more weight, and is defined as

$$S_{xy}^{AA} = \sum_{z \in \mathit{\Gamma}(x) \cap \mathit{\Gamma}(y)} \frac{1}{\log k_z}, \tag{8}$$

(9)Preferential Attachment Index (PA).The mechanism of preferential attachment can be used to generate evolving scale-free networks, where the probability that a new link is connected to the node x is proportional to $k_x$. The probability that this new link will

connect x and y is proportional to $k_x$ $k_y$. Motivated by this mechanism, the corresponding similarity index can be defined as

$$S_{xy}^{PA} = k_x \times k_y, \tag{9}$$

(10)Local Naive Bayes form of CN(LNBCN).

$$S_{xy}^{LNBRA} = \sum_{w \in \Gamma(x) \cap \Gamma(y)} \frac{1}{k_w} (\log R_w + \log s), \tag{10}$$

where $s = \frac{P(A_0)}{P(A_1)}$,$A_0$ and $A_1$ is the class variables of connection and disconnection respectively,and $R_w$ is the role function of node w.
(11)Local Naive Bayes form of RA(LNBRA).

$$S_{xy}^{LNBRA} = |\Gamma(x) \cap \Gamma(y)| \log s + \sum_{w \in \Gamma(x) \cap \Gamma(y)} \log R_w, \tag{11}$$

where $s = \frac{P(A_0)}{P(A_1)}$,$A_0$ and $A_1$ is the class variables of connection and disconnection respectively,and $R_w$ is the role function of node w.
(12)Leicht-Holme-Newman Index (LHN2). This index is a variant of the Katz index. Based on the concept that two nodes are similar if their immediate neighbors are themselves similar, one obtains a self-consistent matrix formulation as

$$S_{xy}^{LHNII} = 2m\lambda_1 D^{-1} (I - \frac{\phi A}{\lambda_1})^{-1} D^{-1}, \tag{12}$$

where D is the degree matrix with $D_{xy}$=$\lambda_{xy} k_x$ and $\phi$ (0<$\phi$<1) is a free parameter. (13)Average Commute Time (ACT).Denote by m(x, y) the average number of steps required by a random walker starting from node x to reach node y, the average commute time between x and y is

$$S_{xy}^{ACT} = \frac{1}{l_{xx}^{+} + l_{yy}^{+} + l_{zz}^{+}}, \tag{13}$$

(14)Cosine based on $L^{+}$. This index is an inner-product-based measure.And the cosine similarity is defined as the cosine of the node vectors, namely

$$S_{xy}^{Cos^{+}} = \frac{v_x^T v_y^T}{|v_x| \cdot |v_y|}, \tag{14}$$

(15)Random Walk with Restart (RWR). This index is a direct application of the PageRank algorithm, and it is defined as

$$S_{xy}^{RWR} = q_{xy}q_{yx}, \tag{15}$$

where $q_{xy}$ is the probability this random walker locates at node y from node x in the steady state.

(16)Local Random Walk (LRW). To measure the similarity between nodes x and y, a random walker is initially put on node x and thus the initial density vector $\vec{\pi}_{(0)}=\vec{e}_x$.The LRW index at time step t is thus defined as

$$S_{xy}^{LRW}(t) = q_x\pi_{xy}(t) + q_y\pi_{yx}(t), \tag{16}$$

where q is the initial configuration function.

(17)Superposed Random Walk (SRW). Similar to the RWR index, Liu and Lü proposed the SRW index, where the random walker is continuously released at the starting point, resulting in a higher similarity between the target node and the nodes nearby. The mathematical expression reads

$$S_{xy}^{SRW}(t) = \sum_{\iota=1}^{t}[q_x\pi_{xy}(\iota) + q_y\pi_{yx}(\iota)], \tag{17}$$

where t denotes the time steps.

(18)Matrix Forest Index (MFI). This index is defined as

$$S_{xy}^{MFI} = (I + L)^{-1}, \tag{18}$$

where the similarity between x and y can be understood as the ratio of the number of s-panning rooted forests such that nodes x and y belong to the same tree rooted at x to all spanning rooted forests of the network.

(19)CN based on transferring similarity(TSCN).This method is CN Index with the transferring similarity,and the similarity is defined as

$$S_{xy}^{Tr} = \epsilon\sum_{v} S_{xv}^{CN}S_{vy}^{Tr} + S_{xy}^{CN}, \tag{19}$$

where $S_{xy}^{Tr}$ is the transferring similarity.

We report the link prediction algorithms' robustness versus their $AUC$ in Fig. S1-S4. We can see that the LRW, SRW, RA and LNBRA have the highest $AUC$. However, their

$R^-$ varies significantly. RA and LNBRA, though have high $AUC$, their $R^-$ is very low, indicating they are very sensitive to the noisy links in the network. On the other hand, LRW and SRW have almost as high robustness as the PA method which only uses node degree for link prediction and thus is very little affected by noise. However, when $R^+$ and $R^e$ are considered, the methods with high $AUC$ tend to have low $R^+$ and $R^e$. This indicate that in these cases, one has to sacrifice the some $AUC$ in order to improve the robustness of the prediction results. The detailed values for $AUC$, $R^+$, $R^-$, $R^e$ are reported in the Table S2 and S3.

## II. SUPPLEMENTARY FIGURES

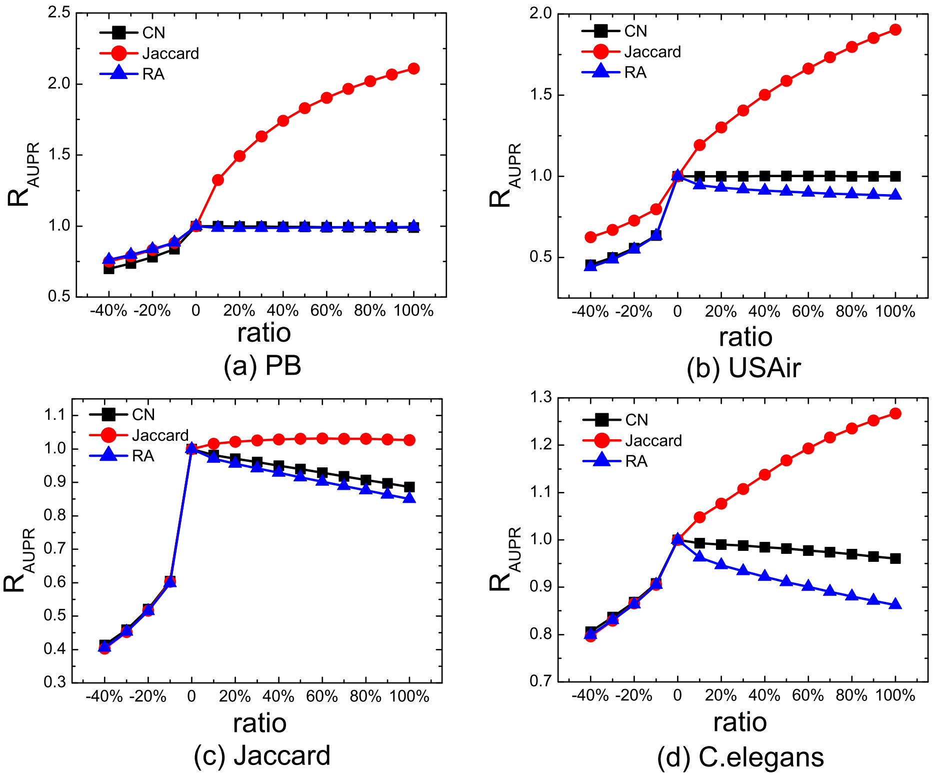


**Figure S1.** The dependence of the robustness of the algorithms ($R_{AUPR}$) on the fraction of missing and noisy links in four real-world networks..

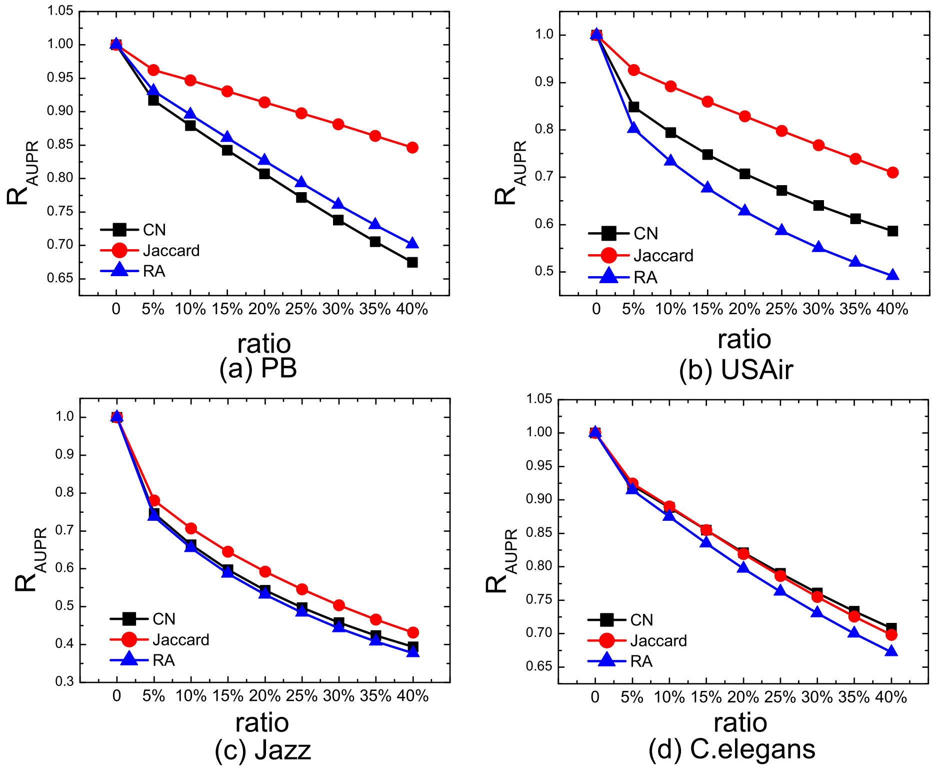


**Figure S2.** The dependence of the robustness of the algorithms ($R_{AUPR}$) on the fraction of rewired links in four real-world networks.

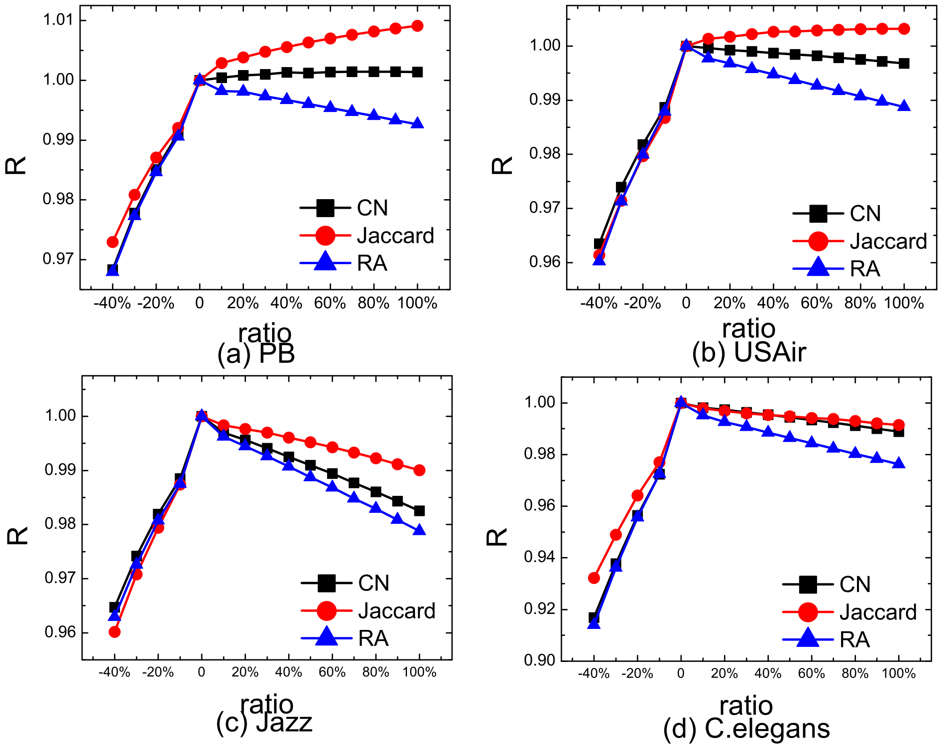


**Figure S3**. The dependence of the robustness of the algorithms $R$ on the fraction of missing and noisy links in four real-world networks. The training set ratio in this figure is 80%.

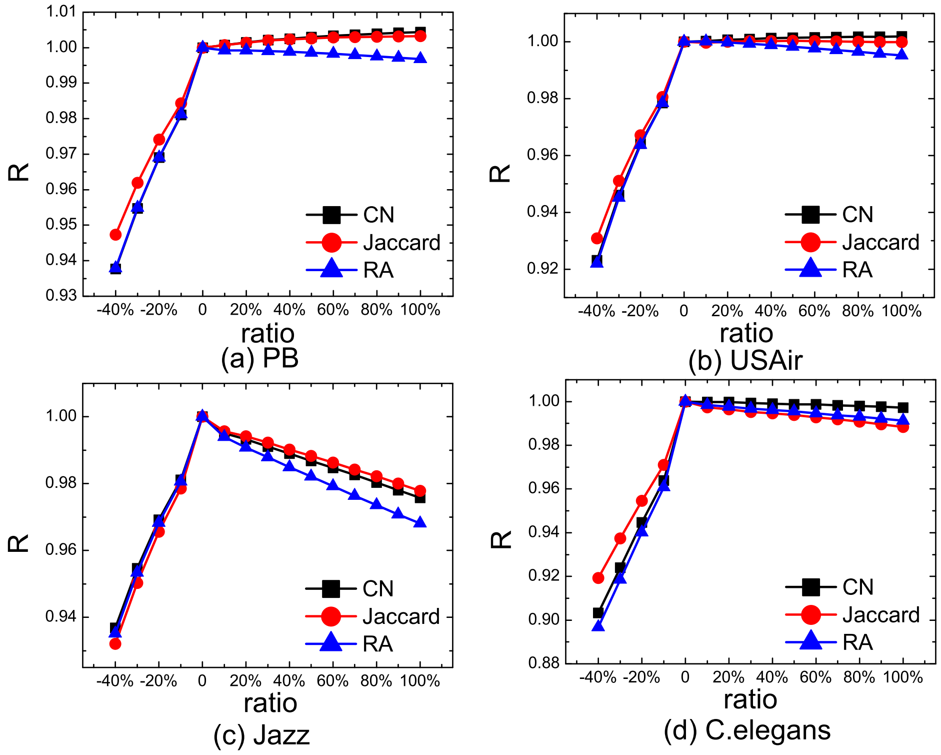


**Figure S4**. The dependence of the robustness of the algorithms $R$ on the fraction of missing and noisy links in four real-world networks. The training set ratio in this figure is 50%.

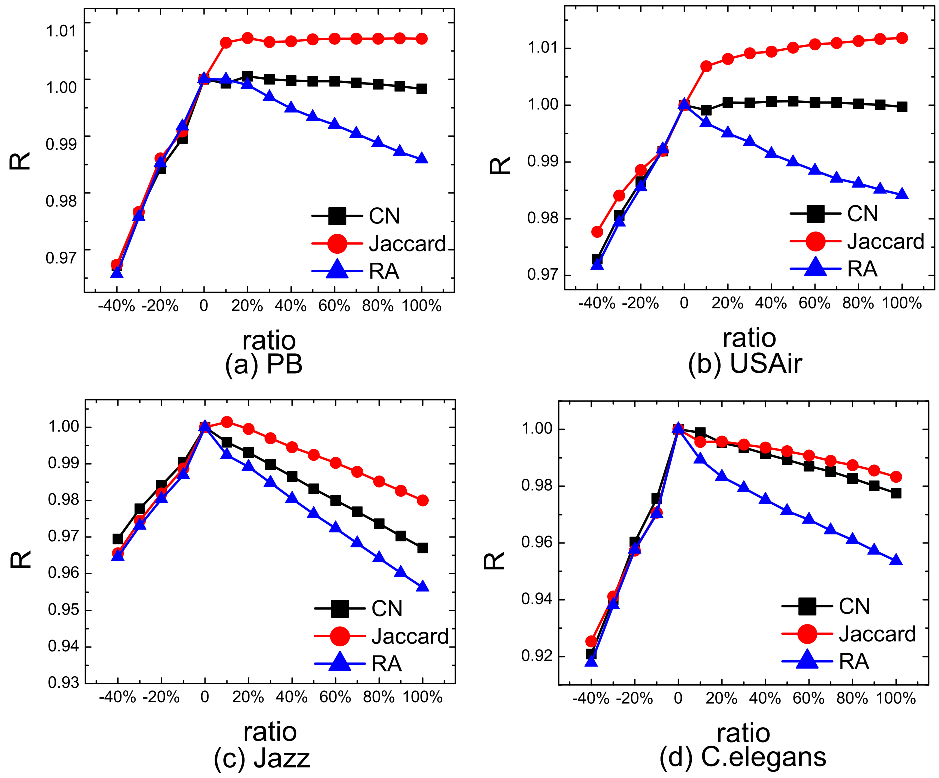


**Figure S5**. the dependence of the robustness of the algorithms $R$ on the fraction of missing and noisy links in four real-world networks. The results are obtained with the 10-fold cross validation.

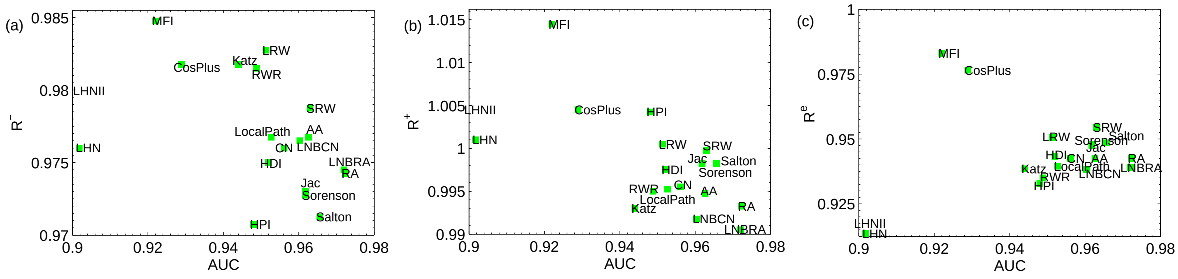


**Figure S6.** The link prediction algorithms' robustness versus their $AUC$ when applied to the Jazz network.

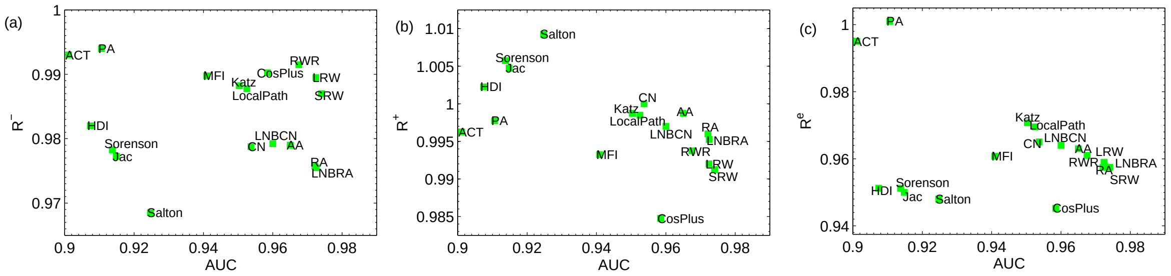


**Figure S7.** The link prediction algorithms' robustness versus their $AUC$ when applied to the USAir network.

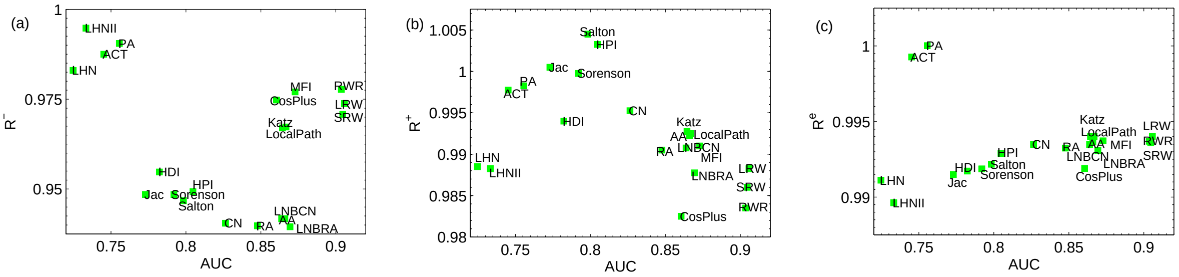


**Figure S8.** The link prediction algorithms' robustness versus their $AUC$ when applied to the C. elegans network.

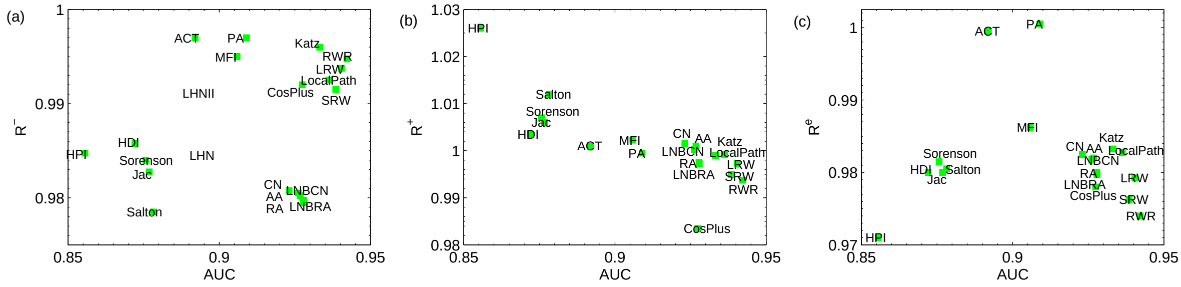


**Figure S9.** The link prediction algorithms' robustness versus their $AUC$ when applied to the PB network.

## III. SUPPLEMENTARY TABLES

**Table S1.** The robustness of link prediction algorithms in ten real networks. $R^-$, $R^+$ and $R^e$ are respectively the robustness of the algorithms with missing links, noisy links and swapped links. The fraction of changed links here is 40%. The highest value for each network is highlighted in bold.

| | $AUC$ | | | $R^-$ | | | $R^+$ | | | $R^e$ | | |
|---|---|---|---|---|---|---|---|---|---|---|---|---|
| Network | $CN$ | $Jaccard$ | $RA$ | $CN$ | $Jaccard$ | $RA$ | $CN$ | $Jaccard$ | $RA$ | $CN$ | $Jaccard$ | $RA$ |
| Dolphins | 0.794 | 0.793 | **0.797** | **0.8903** | 0.8878 | 0.8897 | **0.9953** | 0.9935 | 0.9931 | 0.9123 | 0.9083 | **0.9135** |
| Word | **0.680** | 0.623 | 0.678 | 0.9384 | **0.9661** | 0.9415 | **1.0096** | 0.9993 | 1.0028 | 0.9968 | 0.9905 | **0.9981** |
| Jazz | 0.956 | 0.962 | **0.972** | **0.969** | 0.9652 | 0.9669 | 0.9942 | **0.9975** | 0.9915 | 0.9334 | **0.9404** | 0.9331 |
| E.coli | 0.880 | 0.865 | **0.881** | 0.9321 | **0.9361** | 0.9316 | **1.003** | 1.001 | 1.0006 | **0.9989** | 0.9955 | 0.998 |
| C.elegans | 0.849 | 0.791 | **0.87** | 0.9238 | **0.9343** | 0.9224 | 0.994 | **1.0004** | 0.9882 | **0.9253** | 0.9053 | 0.924 |
| USAir | 0.954 | 0.915 | **0.972** | **0.972** | 0.9702 | 0.9684 | 0.9997 | **1.0055** | 0.9949 | **0.9608** | 0.9433 | 0.9531 |
| Netsci | 0.978 | 0.976 | **0.982** | 0.9227 | **0.9237** | 0.9224 | 0.9992 | **0.9993** | 0.9987 | 0.9071 | 0.9069 | **0.9078** |
| Email | 0.855 | 0.853 | **0.856** | 0.9153 | **0.9164** | 0.9152 | **0.9983** | 0.9979 | 0.9973 | **0.9361** | 0.9339 | 0.9360 |
| PB | 0.923 | 0.877 | **0.928** | 0.9747 | **0.9772** | 0.9731 | 1.002 | **1.0071** | 0.9968 | **0.9796** | 0.9768 | 0.9769 |
| TAP | 0.955 | 0.955 | **0.956** | **0.9582** | 0.958 | 0.958 | 0.9994 | **0.9998** | 0.9994 | 0.9575 | **0.9588** | 0.9582 |

**Table S2.** The *AUC* of link prediction algorithms on four real networks. The three highest values in each column are marked in bold.

| | *Jazz* | *USAir* | *C.elegans* | *PB* |
|---|---|---|---|---|
| CN | 0.9561 | 0.9537 | 0.8264 | 0.9231 |
| Jaccard | 0.9618 | 0.9148 | 0.7730 | 0.8768 |
| RA | **0.9723** | 0.9721 | 0.8477 | 0.9278 |
| LocalPath | 0.9527 | 0.9525 | 0.8668 | 0.9363 |
| Katz | 0.9440 | 0.9503 | 0.8644 | 0.9332 |
| Salton | 0.9656 | 0.9247 | 0.7983 | 0.8782 |
| Sorenson | 0.9618 | 0.9137 | 0.7920 | 0.8757 |
| HDI | 0.9520 | 0.9074 | 0.7823 | 0.8721 |
| HPI | 0.9480 | 0.8816 | 0.8047 | 0.8556 |
| LHN | 0.9018 | 0.7771 | 0.7247 | 0.7631 |
| AA | 0.9626 | 0.9650 | 0.8660 | 0.9268 |
| PA | 0.7697 | 0.9106 | 0.7556 | 0.9090 |
| LNBCN | 0.9603 | 0.9600 | 0.8638 | 0.9262 |
| LNBRA | **0.9720** | **0.9725** | 0.8694 | 0.9279 |
| LHNII | 0.8996 | 0.7694 | 0.7333 | 0.7608 |
| ACT | 0.7976 | 0.9011 | 0.7452 | 0.8920 |
| CosPlus | 0.9289 | 0.9585 | 0.8605 | 0.9274 |
| RWR | 0.9488 | 0.9675 | **0.9036** | **0.9422** |
| LRW | 0.9513 | **0.9724** | **0.9057** | **0.9404** |
| SRW | **0.9630** | **0.9740** | **0.9046** | **0.9385** |
| MFI | 0.9220 | 0.9409 | 0.8728 | 0.9059 |
| TSCN | 0.5119 | 0.6033 | 0.5109 | 0.4953 |

**Table S3.** The robustness index of link prediction algorithms on four real networks. $R^{-}$, $R^{+}$ and $R^{e}$ represent the algorithm robustness for the link removing, link adding and link reshuffling scenarios, respectively. The proportion of changed links is set as 40% for all three scenarios. The three highest values in each column are marked in bold.

| | *Jazz* | | | *USAir* | | | *C.elegans* | | | *PB* | | |
|---|---|---|---|---|---|---|---|---|---|---|---|---|
| Method | $R^{-}$ | $R^{+}$ | $R^{e}$ | $R^{-}$ | $R^{+}$ | $R^{e}$ | $R^{-}$ | $R^{+}$ | $R^{e}$ | $R^{-}$ | $R^{+}$ | $R^{e}$ |
| CN | 0.9690 | 0.9942 | 0.9334 | 0.9720 | 0.9997 | 0.9608 | 0.9238 | 0.9940 | 0.9253 | 0.9747 | 1.0020 | 0.9796 |
| Jaccard | 0.9652 | 0.9975 | 0.9404 | 0.9702 | 1.0055 | 0.9433 | 0.9343 | **1.0004** | 0.9053 | 0.9772 | 1.0071 | 0.9768 |
| RA | 0.9669 | 0.9915 | 0.9331 | 0.9684 | 0.9949 | 0.9531 | 0.9224 | 0.9882 | 0.9240 | 0.9731 | 0.9968 | 0.9769 |
| LocalPath | 0.9705 | 0.9939 | 0.9312 | 0.9842 | 0.9983 | 0.9663 | 0.9583 | 0.9906 | 0.9324 | 0.9914 | 0.9989 | 0.9808 |
| Katz | 0.9764 | 0.9911 | 0.9303 | 0.9849 | 0.9986 | **0.9676** | 0.9581 | 0.9909 | 0.9323 | 0.9946 | 0.9986 | 0.9814 |
| Salton | 0.9629 | 0.9976 | 0.9399 | 0.9594 | **1.0109** | 0.9413 | 0.9320 | **1.0052** | 0.9115 | 0.9723 | **1.0145** | 0.9777 |
| Sorenson | 0.9649 | 0.9976 | 0.9392 | 0.9712 | 1.0068 | 0.9451 | 0.9342 | 0.9995 | 0.9082 | 0.9787 | 1.0083 | 0.9789 |
| HDI | 0.9678 | 0.9969 | 0.9346 | 0.9759 | 1.0025 | 0.9451 | 0.9419 | 0.9926 | 0.9068 | 0.9811 | 1.0040 | 0.9774 |
| HPI | 0.9625 | 1.0048 | 0.9219 | 0.9696 | **1.0471** | 0.9302 | 0.9348 | **1.0040** | 0.9197 | 0.9801 | **1.0316** | 0.9669 |
| LHN | 0.9691 | 1.0013 | 0.8996 | **1.0242** | 1.0029 | 0.8867 | 0.9767 | 0.9855 | 0.9001 | **1.0241** | 0.9988 | 0.9523 |
| AA | 0.9700 | 0.9934 | 0.9342 | 0.9720 | 0.9982 | 0.9585 | 0.9250 | 0.9903 | 0.9281 | 0.9740 | 1.0010 | 0.9801 |
| PA | **0.9858** | 0.9991 | **1.0000** | **0.9925** | 0.9971 | **1.0009** | **0.9877** | 0.9976 | **1.0004** | 0.9963 | 0.9994 | **1.0007** |
| LNBCN | 0.9698 | 0.9896 | 0.9295 | 0.9726 | 0.9962 | 0.9597 | 0.9252 | 0.9885 | 0.9264 | 0.9744 | 1.0001 | 0.9796 |
| LNBRA | 0.9670 | 0.9879 | 0.9299 | 0.9680 | 0.9942 | 0.9528 | 0.9221 | 0.9848 | 0.9222 | 0.9733 | 0.9965 | 0.9772 |
| LHNII | 0.9746 | **1.0054** | 0.9040 | **1.0195** | **1.0132** | 0.8662 | **0.9928** | 0.9856 | 0.8833 | **1.0296** | **1.0149** | 0.9385 |
| ACT | **0.9816** | 0.9837 | 0.9722 | 0.9911 | 0.9954 | **0.9946** | 0.9841 | 0.9971 | **0.9920** | 0.9963 | 1.0012 | **0.9995** |
| CosPlus | 0.9770 | 1.0053 | 0.9702 | 0.9874 | 0.9811 | 0.9387 | 0.9675 | 0.9778 | 0.9069 | 0.9896 | 0.9798 | 0.9753 |
| RWR | 0.9764 | 0.9928 | 0.9255 | 0.9893 | 0.9922 | 0.9567 | 0.9713 | 0.9795 | 0.9290 | 0.9933 | 0.9922 | 0.9709 |
| LRW | 0.9778 | 0.9999 | 0.9419 | 0.9864 | 0.9902 | 0.9542 | 0.9662 | 0.9854 | 0.9321 | 0.9919 | 0.9967 | 0.9764 |
| SRW | 0.9729 | 0.9995 | 0.9461 | 0.9832 | 0.9891 | 0.9522 | 0.9623 | 0.9826 | 0.9273 | 0.9891 | 0.9937 | 0.9730 |
| MFI | **0.9806** | **1.0172** | **0.9774** | 0.9866 | 0.9914 | 0.9556 | 0.9702 | 0.9887 | 0.9282 | 0.9937 | 1.0025 | 0.9841 |
| TSCN | 0.9428 | **1.0104** | **0.9891** | 0.8321 | 1.0035 | 0.8921 | **1.0365** | 0.9569 | **1.0557** | **0.9970** | 0.9980 | **0.9978** |

[1] Lü, L., Jin, C. H. & Zhou, T. Similarity index based on local paths for link prediction of complex networks. *Phys. Rev. E* **80**, 046122 (2009).

[2] Katz, L. A new status index derived from sociometric analysis. *Psychometrika* **18**, 39 (1953).

[3] Salton, G. & McGill, M. J. *Introduction to Modern Information Retrieval.* (1983).

[4] Sorensen, T. A method of establishing groups of equal amplitude in plant sociology based on similarity of species content and its application to analyses of the vegetation on Danish commons. *Biol. Skr.* **5**,1 (1948).

[5] Zhou, T., Lü, L. & Zhang, Y. C. Predicting missing links via local information. *Eur. Phys. J. B.* **71**, 623 (2009).

[6] Ravasz, E., Somera, A. L., Mongru, D. A., Oltvai, Z. N. & Barabási,A. L. Hierarchical organization of modularity in metabolic networks, *Science* **297**, 1551-1555 (2002).

[7] Leicht, E. A., Holme, P. & Newman, M. E. Vertex similarity in networks. *Phys. Rev. E* **73**, 026120 (2006).

[8] Adamic, L. A. & Adar, E. Friends and neighbors on the Web. *Soc. Networks* **25**, 211-230(2003).

[9] Barabási, A. L. & Albert, R. Emergence of scaling in random networks. *Science* **286**, 509-512 (1999).

[10] Liu, Z., Zhang, Q. M., L, L. & Zhou, T. Link prediction in complex networks: A local naive Bayes model. *Europhys. Lett.* **96**, 48007 (2011).

[11] Klein, D. J., Randić, M. Resistance distance. *J. Math. Chem.* **12**, 81-95 (1993).

[12] Fouss, F., Pirotte, A., Renders, J. M. & Saerens, M. Random-walk computation of similarities between nodes of a graph with application to collaborative recommendation. *IEEE Trans. Knowl. Data. Eng.* **19**,355-369 (2007).

[13] Brin, S. & Page, L. The anatomy of a large-scale hypertextual Web search engine. *Comput. Netw. ISDN Syst* **30**,107-117 (1998).

[14] Liu, W. & Lü, L. Link prediction based on local random walk. *Europhys. Lett.* **89**, 58007 (2010).

[15] Chebotarev, P. & Shamis, E. The matrix-forest theorem and measuring relations in small

social groups. *arXiv* preprint math/0602070 (2006).

[16] Sun, D. *et al.* Information filtering based on transferring similarity. *Phys. Rev. E* **80**, 017101 (2009).
